# RexAB Is Essential for the Mutagenic Repair of Staphylococcus aureus DNA Damage Caused by Co-trimoxazole

**DOI:** 10.1128/AAC.00944-19

**Published:** 2019-11-21

**Authors:** Rebecca S. Clarke, Maya S. Bruderer, Kam Pou Ha, Andrew M. Edwards

**Affiliations:** a MRC Centre for Molecular Bacteriology and Infection, Imperial College London, London, United Kingdom

**Keywords:** reactive oxygen species, SOS response, DNA damage, DNA repair, SOS system, *Staphylococcus aureus*, co-trimoxazole, oxidative damage

## Abstract

Co-trimoxazole (SXT) is a combination therapeutic that consists of sulfamethoxazole and trimethoprim that is increasingly used to treat skin and soft tissue infections caused by methicillin-resistant Staphylococcus aureus (MRSA). However, the use of SXT is limited to the treatment of low-burden, superficial S. aureus infections and its therapeutic value is compromised by the frequent emergence of resistance. As a first step toward the identification of approaches to enhance the efficacy of SXT, we examined the role of bacterial DNA repair in antibiotic susceptibility and mutagenesis. We found that mutants lacking the DNA repair complex RexAB had a modest SXT MIC that was 2-fold lower than that seen with wild-type strains but were killed 50-fold to 5,000-fold more efficiently by the combination antibiotic at the breakpoint concentration. SXT-mediated DNA damage occurred via both thymidine limitation and the generation of reactive oxygen species and triggered induction of the SOS response in a RexAB-dependent manner. SOS induction was associated with a 50% increase in the mutation rate, which may contribute to emergence of resistant strains during SXT therapy. In summary, this work determined that SXT caused DNA damage in S. aureus via both thymidine limitation and oxidative stress and that the damage was repaired by the RexAB complex, leading to induction of the mutagenic SOS response. Small-molecule inhibitors of RexAB could therefore have therapeutic value by increasing the efficacy of SXT and decreasing the emergence of drug resistance during treatment of infections caused by S. aureus.

## Abstract (precis)

Co-trimoxazole (SXT) is a combination therapeutic that consists of sulfamethoxazole and trimethoprim that is increasingly used to treat skin and soft tissue infections caused by methicillin-resistant Staphylococcus aureus (MRSA). However, the use of SXT is limited to the treatment of low-burden, superficial S. aureus infections and its therapeutic value is compromised by the frequent emergence of resistance.

## INTRODUCTION

Staphylococcus aureus is responsible for a wide spectrum of diseases ranging from superficial skin infections to life-threatening bacteremia, endocarditis, and toxic shock syndrome (1). While β-lactam antibiotics such as oxacillin are first-choice therapeutics for S. aureus infections, the prevalence of skin and soft tissue infections (SSTIs) caused by methicillin-resistant S. aureus (MRSA) strains has necessitated the use of second-line therapeutics such as co-trimoxazole (SXT) (2, 3). SXT has several desirable properties, including low cost, the availability of both oral and intravenous formulations, and low host toxicity, making it an appealing treatment option (4).

SXT is a combination of two antibiotics, trimethoprim (TMP) and sulfamethoxazole (SMX), in a 1:5 ratio, and the two antibiotics target sequential steps in the tetrahydrofolate biosynthetic pathway (5). SMX inhibits dihydropteroate synthetase (DHPS), preventing the production of dihydropteroic acid, while TMP binds and inhibits dihydrofolate reductase (DHFR), blocking the conversion of dihydrofolic acid to tetrahydrofolate (6). Since the production of tetrahydrofolate is essential for the biogenesis of thymidine, purines, and some amino acids, bacteria exposed to SXT experience disrupted metabolic activity and stalled DNA replication (7).

Previous studies have reported that TMP-induced thymidine depletion contributes to bacterial cell death through stalling of DNA replication forks, which, together with the continued initiation of replication, results in DNA damage (8). However, one of the limitations of the use of SXT as an antibiotic is that S. aureus can bypass SXT-mediated metabolic blockage by utilizing exogenous thymidine released from damaged tissues (9). It is therefore hypothesized that the presence of thymidine at infection sites results in a reduction of the efficacy of SXT treatment in patients (10–12). As a consequence, SXT is used only for low-burden superficial staphylococcal infections.

In addition to SXT-mediated DNA damage occurring via stalled replication forks, recent work with Escherichia coli has indicated that production of reactive oxygen species (ROS) and maladaptive DNA repair contribute to the bactericidal activity of this antibiotic (13). However, it is not clear if this is specific to E. coli or represents a general mechanism of bactericidal activity for many different bacteria.

SXT-mediated DNA damage triggers the SOS response, which is an inducible repair system that enables bacteria to survive genotoxic stress (14, 15). Regulation of the SOS response is highly conserved and occurs primarily via LexA, a transcriptional repressor of the SOS regulon (16). When DNA damage occurs, RecA binds to single-stranded DNA (ssDNA) at the lesion site to form a nucleoprotein filament which (i) facilitates DNA strand invasion that initiates repair by homologous recombination and (ii) induces the autocleavage of LexA, resulting in derepression of the SOS regulon (17). It is believed that RecF assists RecA binding to ssDNA gaps from single-nucleotide lesions in S. aureus (18). However, double-strand breaks (DSBs) are recognized and processed by the AddAB complex (known as RexAB in S. aureus), generating an ssDNA overhang through the enzyme's helicase/exonuclease activity. This ssDNA strand then serves as the substrate for RecA binding and activation of the SOS response (19).

The SOS regulon of S. aureus consists of 16 LexA-regulated genes (17), including *recA* and *lexA* as well as *umuC*, which encodes a low-fidelity DNA polymerase. UmuC catalyzes translesion DNA synthesis but lacks proofreading ability and permits DNA replication across unresolved lesions, which often introduces mutations. Such mutagenic DNA repair may be advantageous to the pathogen by conferring resistance to antibiotics or adaptation to host stresses (17, 20). For example, mutations in the gene encoding DHFR resulted in reduced enzyme activity and were associated with TMP resistance, with MICs of 8 to >512 μg ml^−1^ (21). Similarly, mutations in the gene encoding DHPS resulted in SMX resistance, with MICs of ≥256 μg ml^−1^ (22). Therefore, exposure of S. aureus to SXT at concentrations that do not kill the pathogen may promote the emergence of resistance via induction of the mutagenic SOS response.

The prevalence of infections caused by drug-resistant pathogens necessitates new therapeutic options. However, given the lack of investment into the development of new antibiotics, there is increasing interest in the development of therapeutics that would enhance the efficacy of existing antibacterial drugs or bypass resistance (23, 24). However, such an approach requires a thorough understanding of how antibiotics function and the mechanisms used by bacteria to survive exposure to antibacterial drugs. Therefore, the aim of this work was to identify the mechanisms by which SXT damages S. aureus DNA, the repair systems that this pathogen uses to withstand this damage, and the consequences of repair for the emergence of resistance.

## RESULTS

### Inactivation of *rexB* sensitizes S. aureus to SXT.

To investigate the role of bacterial DNA repair in the susceptibility of S. aureus to SXT, a panel of S. aureus transposon mutants were subjected to MIC determinations and disc diffusion susceptibility tests of antibiotics, including SXT and its constituent antibiotics, TMP and SMX. The mutants each lacked a component of DNA repair (*rexA*, *rexB*, *recF*, *umuC*, or *dinB*) previously shown to contribute to the repair of damage caused by the quinolone antibiotic ciprofloxacin or acted as controls (20). Mutants in two distinct genetic backgrounds were used in this study, namely, SH1000, a well-established laboratory strain, and JE2, a community-associated methicillin-resistant S. aureus (CA-MRSA) strain of the USA300 lineage that causes severe skin infections that are often treated with SXT (3, 25).

**FIG 1:**
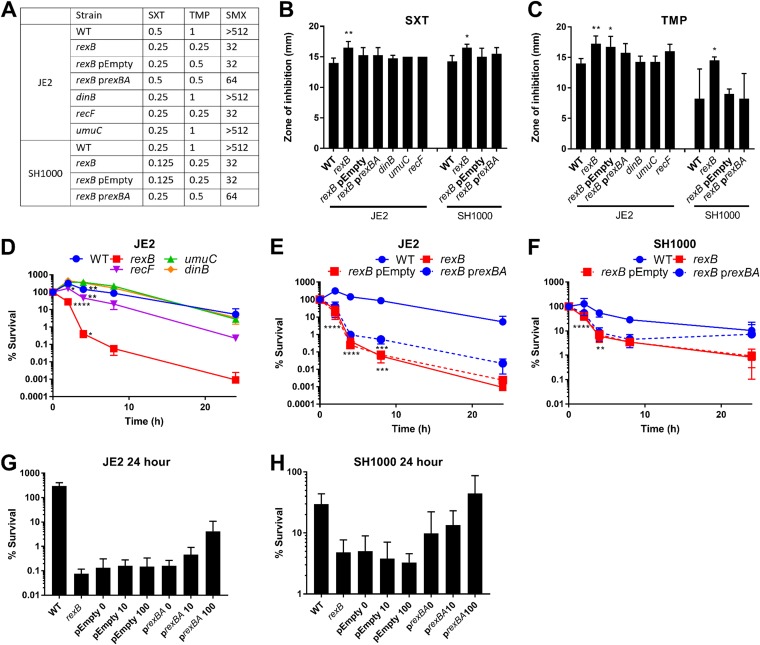
Mutants defective for DNA repair have increased susceptibility to SXT and its constituent antibiotics TMP and SMX. (A) MIC values for SXT, TMP, and SMX for wild-type S. aureus JE2 and SH1000 and mutants defective for DNA damage repair in TSB. Data represent median values (*n* = 3). (B and C) Zones of growth inhibition of WT JE2 and SH1000 S. aureus strains or damage repair mutants from paper discs impregnated with 2.5 μg SXT (B) or TMP (C) after 16 h of incubation on TSA plates. Graphs represent means ± SD (*n* = 4). Values that are significantly different from the WT values for each antibiotic exposure were identified by one-way ANOVA. (D, E, and F) Time course survival assays of S. aureus JE2 (D and E) and SH1000 (F) and their derived DNA damage repair mutants during incubation in TSB supplemented with 4 μg ml^−1^ SXT. Data are split between panels D and E for clarity. Percent survival at each time point was calculated relative to the starting inoculum of ∼1 × 10^8^ CFU ml^−1^. Graphs represent means ± SD (*n* = 3). (G and H) Survival of S. aureus JE2 (G) and SH1000 (H) at 24 h in TSB supplemented with 4 μg ml^−1^ SXT. Survival data include the WT strain, *rexB* mutants, and *rexB* mutants transformed with pEmpty or p*rexBA*, which were grown to the stationary phase in the presence of either 0, 10 or 100 ng/ml AHT to regulate *rexBA* expression. Graphs represent means ± SD (*n* = 4). Values that were significantly different from the WT were identified by two-way ANOVA corrected for multiple comparisons using the Dunnett method. *, *P ≤* 0.05; **, *P ≤* 0.01; ***, *P ≤* 0.001; ****, *P ≤* 0.0001.

The MIC values for both the SH1000 and JE2 wild-type (WT) strains indicated susceptibility to SXT and TMP (<2 μg ml^−1^) but resistance to SMX (>512 μg ml^−1^) on the basis of EUCAST breakpoints for S. aureus (EUCAST, 2019) (Fig. 1A). Despite the resistance to SMX, the sulfonamide appeared to synergize with TMP, since the MIC for SXT was 2-fold lower than that of TMP for JE2 and 4-fold lower for SH1000, which is in keeping with results of previous work (26). Disruption of *rexB* in both the JE2 and SH1000 strains lowered the MIC of SXT and TMP by 2-fold to 4-fold and that of SMX by at least 16-fold, while complementation of the *rexB* mutant led to a doubling in the MICs for SXT for both JE2 and SH1000 relative to the mutant strain containing the empty plasmid control (Fig. 1A). The mutant lacking *recF* in the JE2 strain was also more susceptible than the WT strain to all three antibiotics, but disruption of *dinB* or *umuC* resulted in only slightly increased susceptibility to SXT but not TMP or SMX.

Consistent with the MIC data, disc diffusion assays performed with both the JE2 and SH1000 strains showed that inactivation of *rexB* caused a small but significant increase in the susceptibility of S. aureus to SXT and TMP relative to the WT (Fig. 1B and C). Complementation of the *rexB* mutant (p*rexBA*) reduced susceptibility to a level close to that seen with the WT, confirming the role of the RexAB complex (Fig. 1B and C). In contrast, inactivation of *dinB or umuC* did not significantly increase zone sizes for either antibiotic (Fig. 1B and C). However, the *recF*-deficient mutant showed an increase in susceptibility to TMP in the JE2 strain (Fig. 1C), indicating that both *rexBA* and *recF* contribute to the repair of DNA damage caused by TMP.

The finding that RexAB and RecF reduced the susceptibility of S. aureus to SXT confirmed that DNA repair modulated susceptibility to SXT and prompted us to undertake a screen of mutants with a disrupted copy of *recJ*, *uvrA*, *uvrB*, *nth*, *mutS*, *mutL*, *sbcC*, or *sbcD*, to identify any additional repair processes that might be involved. However, none of these mutants had a SXT MIC that was different from that of the wild type. In summary, mutants lacking *rexA*, *rexB*, or *recF* had minimally but statistically significantly increased levels of susceptibility to the growth-inhibitory activity of SXT and each of its two components, TMP and SMX.

Despite the increased susceptibility of mutants lacking *rexA*, *rexB*, or *recF* to SXT seen in MIC and zone-of-inhibition assays, the differences observed were minimal and of questionable clinical relevance. Therefore, we next investigated the contribution of DNA repair to the survival of S. aureus (∼10^8^ CFU ml^−1^) exposed to the breakpoint concentration of the antibiotic (4 μg ml^−1^) (EUCAST, 2019). Despite the reported bactericidal activity of SXT, there was only a 20-fold reduction in survival over 24 h for the JE2 strain (Fig. 1D), indicating a relatively high level of tolerance of SXT at this concentration (26, 27). Similar levels of survival were seen for mutants lacking *umuC* or *dinB* in the JE2 background (Fig. 1D). In contrast, survival of the *recF* mutant was reduced by >20-fold, relative to the WT, indicating increased susceptibility. Most notably, however, the *rexB* mutant showed an approximately 5,000-fold reduction in CFU counts relative to the WT at 24 h. Complementation of the *rexB* mutant increased survival 10-fold relative to a plasmid-only control (Fig. 1E). Subsequent experiments performed with the SH1000 strain confirmed a role for *rexBA* in modulating the susceptibility of S. aureus to SXT. There was a 12-fold-greater loss of CFU counts with the *rexB* mutant than with the WT (Fig. 1F). However, similarly to what was seen for the MIC assays, complementation of the *rexB* mutant with p*rexBA* restored survival levels to close to those of the WT (Fig. 1F).

For both the JE2 and SH1000 strains, complementation of the *rexAB*::Tn mutants employed an anhydrotetracycline hydrochloride (AHT)-inducible promoter system that enabled control of *rexAB* expression, enabling dose-response studies to be undertaken. In the absence of AHT, the level of bacterial survival was similar to that seen for the *rexAB*::Tn mutant after 24 h. However, the addition of 10 or 100 μg ml^−1^ AHT resulted in a dose-dependent increase in survival, providing further evidence that RexAB promotes the survival of S. aureus strains exposed to co-trimoxazole at 4 μg ml^−1^ (Fig. 1G and H). It is unclear why the complementation system fully restored the WT phenotype in the SH1000 strain but did so only partially in the JE2 strain (Fig. 1G and H). However, it is not uncommon for complementation of S. aureus to confer a stronger or weaker phenotype than the WT, including when AHT-inducible systems such as p*itet* are used (28).

In summary, despite small differences in the susceptibility of wild-type and *rexB* mutant to the growth-inhibitory activity of SXT (as defined by MIC values), the DNA repair mutant was significantly more susceptible to the combination antibiotic when used at the breakpoint (8× wild-type MIC). These findings indicate that SXT causes DNA double-strand breaks in S. aureus when used at the breakpoint concentration, the repair of which by RexAB significantly promotes staphylococcal survival during exposure to the antibiotic.

### Thymidine limitation contributes to SXT-mediated DNA damage.

Inhibition of folate biosynthesis by SXT results in abrogation of endogenous thymidine biosynthesis (29), which could provide an explanation for the DNA damage phenotype described above (Fig. 1). In support of this, previous studies have reported that the *in vitro* inhibitory activity of TMP and SXT is inversely correlated with the availability of thymidine in the culture medium (10, 30). The presence of exogenous thymidine allows S. aureus to continue with canonical DNA synthesis and to allow effective DNA repair to occur, despite SXT-mediated inhibition of tetrahydrofolate production (13).

**FIG 2:**
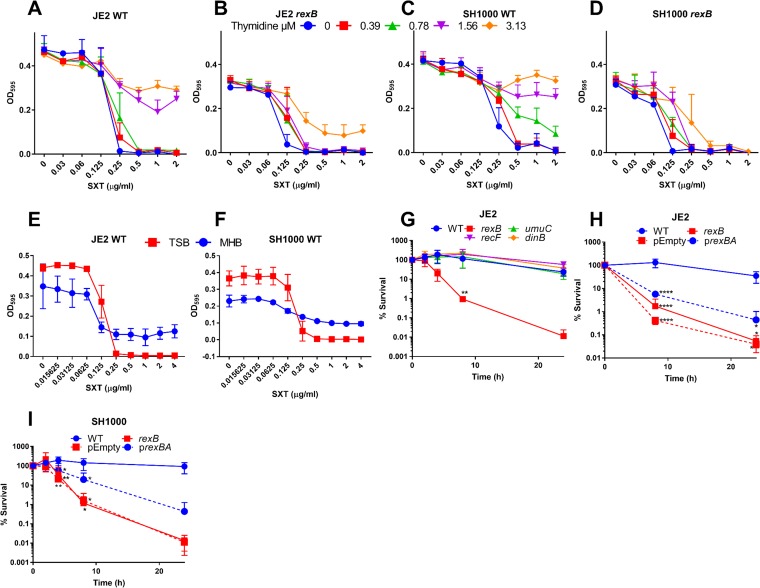
Mutants defective for *rexB* have increased susceptibility to SXT under thymidine-rich conditions. (A to D) Growth (OD_600_) of S. aureus JE2 WT (A), a JE2-derived *rexB* mutant (B), SH1000 WT (C), and a SH1000-derived *rexB* mutant (D) after 16 h of static incubation at 37°C in the presence of doubling concentrations of SXT in TSB supplemented with 0 to 3.13 μM thymidine as indicated. Graphs represent means ± SD (*n* = 3). (E and F) Growth (OD_595_) of S. aureus JE2 WT (E) and SH1000 WT (F) in TSB and MHB supplemented with doubling dilution concentrations of SXT. Graphs represent means ± SD (*n* = 3). (G to I) Time course survival assays of S. aureus JE2 WT or mutants defective for DNA damage repair (G); S. aureus JE2 WT, JE2 *rexB* mutant only, or JE2 *rexB* mutant transformed with empty vector (pEmpty) or complemented mutant (p*rexBA*) (H); and SH1000 WT, *rexB* mutant only, or *rexB* mutant transformed with pEmpty or p*rexBA* (I). For panels G to I, all strains were incubated in MHB containing 4 μg ml^−1^ SXT. Percent survival at each time point was calculated relative to the starting inoculum of ∼1 × 10^8^ CFU ml^−1^. Graphs represent means ± SD (*n* = 3). Values that were significantly different from the WT were determined by two-way ANOVA corrected for multiple comparisons using the Dunnett method. *, *P ≤* 0.05; **, *P ≤* 0.01; ***, *P ≤* 0.001; ****, *P ≤* 0.0001.

While the precise compositions of media can differ between manufacturers and from batch to batch, tryptic soy broth (TSB) contains a low concentration of thymidine (reported previously to be 0.04 μg ml^−1^, i.e., 0.25 μM [30]). To investigate the influence of exogenous thymidine on SXT activity in our assays, the growth of WT and *rexB* mutant strains was determined in TSB supplemented with doubling dilutions of SXT and thymidine via checkerboard assays (Fig. 2A to D). As expected, the presence of high concentrations of thymidine enabled growth of WT strains of both JE2 and SH1000 at higher concentrations of SXT (30) (Fig. 2A and C).

Given the thymidine-dependent activity of SXT, different culture medium compositions can greatly affect antibiotic susceptibility (10, 30). For example, Mueller-Hinton broth (MHB), the gold standard medium for antibiotic susceptibility testing, contains 0.46 μg ml^−1^ thymidine compared to 0.04 μg ml^−1^ thymidine in TSB, representing a difference which can greatly affect susceptibility testing (30). Therefore, susceptibility tests were undertaken using MHB to compare with those described above using TSB. Consistent with the checkerboard data and the literature (30), the efficacy of SXT in MHB was reduced relative to that in TSB, with detectable bacterial growth of both WT strains observed even at high concentrations of SXT, most likely due to the increased levels of thymidine (Fig. 2E and F).

To explore the effect of the presence of exogenous thymidine on the bactericidal activity of SXT, time course killing assays were undertaken using MHB. As expected from the MIC assays, and in contrast to assays done in TSB (Fig. 1D), there was almost no killing of the S. aureus JE2 WT strain by SXT (4 μg ml^−1^) in MHB over 24 h (Fig. 2G). Similarly high levels of survival were observed for the *dinB*, *umuC*, and *recF* mutants (Fig. 2G). In contrast, the rate of survival of the *rexB* mutant in MHB containing SXT was >2,000-fold lower than that of the WT, despite the presence of exogenous thymidine (Fig. 2G). Similarly to TSB, complementation of the *rexB* mutant (p*rexBA*) significantly promoted survival in MHB containing SXT relative to the results seen with a plasmid-only control (Fig. 2H). Similar results were seen for the SH1000 strain, with the WT strain surviving in MHB containing SXT (4 μg ml^−1^), while the viability of the *rexB* mutant was reduced >6,000-fold relative to the inoculum (Fig. 2I). As seen with the JE2 strain, complementation of the SH1000 *rexB* mutant significantly promoted survival (>10-fold) (Fig. 2I).

Combined, these data demonstrate that the RexAB DNA repair complex is required for the survival of S. aureus exposed to SXT, even when thymidine is available, suggesting that thymidine limitation is not the only mechanism by which this combination antibiotic damages bacterial DNA.

### Inactivation of *rexB* sensitizes S. aureus to SXT-mediated growth inhibition but not to killing under anaerobic conditions.

It was recently reported that TMP exposure increases the production of reactive oxygen species (ROS) by E. coli, which, together with incomplete DNA repair, contributes to bacterial cell death (13). Therefore, we hypothesized that SXT-mediated DNA damage could occur via stalling of DNA synthesis caused by inhibition of thymidine production and via the generation of ROS.

**FIG 3:**
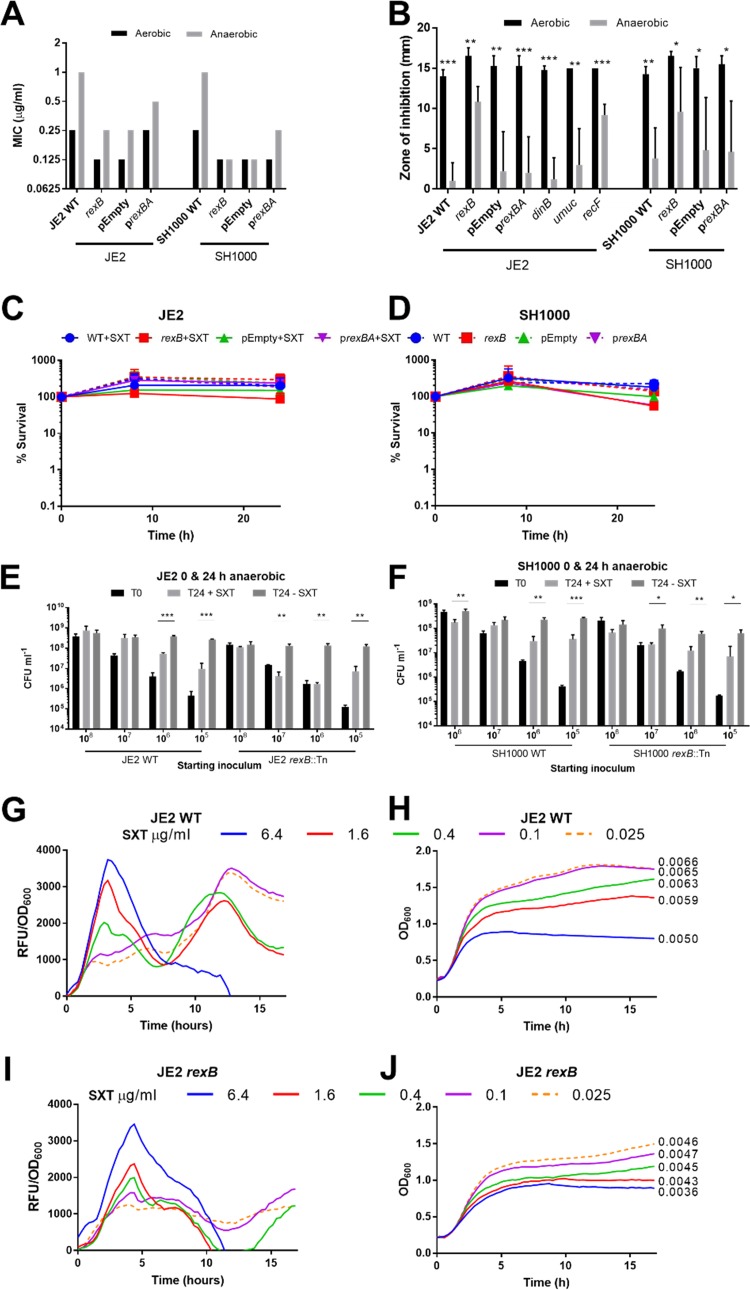
Reactive oxygen species are essential for SXT-mediated killing of S. aureus. (A) MIC of SXT for WT S. aureus JE2 and SH1000 and their derived mutants defective for DNA repair determined in TSB under aerobic or anaerobic conditions. Graphs represent median values (*n* = 3). (B) Zones of inhibition around paper discs containing SXT (2.5 μg) on agar plates inoculated with S. aureus JE2 WT or SH1000 WT and associated DNA repair mutants on TSA after 16 h of incubation under aerobic (black) or anaerobic (gray) conditions. The graph represents means ± SD (*n* = 4). Zones which were significantly different in size between aerobic and anaerobic conditions were determined by a two-tailed Student's *t* test. (C and D) Time course survival assays of S. aureus JE2 (C) and SH1000 (D) WT or *rexB* mutants without or with empty vector (pEmpty) or complemented (p*rexBA*) in TSB with (+SXT) or without 4 μg ml^−1^ SXT, under anaerobic conditions. Percent survival at each time point was calculated relative to the starting inoculum of ∼1 × 10^8^ CFU ml^−1^. Graphs represent means ± SD (*n* = 3). Values that are statistically significantly different from the WT values were determined by two-way ANOVA corrected for multiple comparisons using the Sidak method. (E and F) Survival of S. aureus JE2 (E) and SH1000 (F) WT and *rexB* mutants at 24 h (T24) in TSB with (+SXT) and without (-SXT) 4 μg ml^−1^ SXT under anaerobic conditions. Starting inoculums (T0) ranged from 1 × 10^8^ to 1 × 10^5^ CFU ml^−1^. The graph represents means ± SD (*n* = 4). The statistical significance of the results of comparisons between the levels of growth at T24 in the presence and absence of SXT was determined by multiple two-tailed *t* tests. *, *P ≤* 0.05; **, *P ≤* 0.01; ***, *P ≤* 0.001; ****, *P ≤* 0.0001. (G and H) ROS production by bacteria was detected using the DCF fluorophore for S. aureus JE2 WT (G) or the *rexB* mutant (H) in the presence of SXT. (I and J) RFU data generated by ROS were normalized to RFU/OD_600_ data (I) and OD_600_ data (J) to determine levels of ROS production relative to cell density. Error bars were omitted from the data in panels G to J for clarity. For panels H and J, logarithmic growth rates (ΔOD_600_ min^−1^) for each SXT concentration are shown adjacent to the line graphs.

To examine whether the production of ROS contributes to SXT activity against S. aureus, the susceptibility of WT and DNA repair mutants to the antibiotic was determined under both aerobic and anaerobic conditions in low-thymidine TSB. In the absence of oxygen, the MIC of WT S. aureus was increased 4-fold for both the JE2 and SH1000 strains (Fig. 3A). In contrast, the SXT MIC of the JE2 *rexB* mutant only doubled under anaerobic conditions, while the MIC of the SH1000 *rexB* mutant was unaffected by the presence of oxygen (Fig. 3A). To explore this further, zone-of-inhibition assays were done under aerobic and anaerobic conditions. Zones of inhibition were significantly reduced in the absence of oxygen for all strains and mutants, implying reduced susceptibility (Fig. 3B). However, despite the reduced antibiotic activity of SXT under anaerobic conditions, the *rexBA* mutants were still more susceptible than the corresponding WT, with >10-fold- and 2.5-fold-greater zone sizes for JE2 and SH1000 strains, respectively (Fig. 3B).

The elevated susceptibility of the *rexB* mutant to SXT under both aerobic and anaerobic conditions indicated that thymidine limitation causes double-strand breaks via a process that is promoted by, but not dependent upon, the presence of oxygen.

Next, we assessed the contribution of ROS to the bactericidal activity of SXT. In contrast to assays done in air, none of the strains examined were killed by SXT (4 μg ml^−1^) under anaerobic conditions (Fig. 3C and D). However, while the WT strains were able to grow, albeit minimally, under these conditions, SXT inhibited growth of the *rexB* mutants by >2-fold by 24 h postinoculation (Fig. 3C and D). Complementation of the *rexB* mutant, but not the empty vector, rescued SXT-mediated growth inhibition for both strains at 24 h (Fig. 3C and D). Therefore, SXT exposure caused bacteriostasis in the *rexBA* mutant but not in the WT strain under anaerobic conditions.

We hypothesized that the ability of the WT strains to grow in the presence of an SXT concentration above the MIC (Fig. 3C and D) likely reflected the 1,000-fold-larger inoculum used in time-kill experiments (∼10^8^ CFU ml^−1^) than in the MIC assays (∼10^5^ CFU ml^−1^) (31–33). Since this phenomenon, known as the inoculum effect, has been reported to affect the co-trimoxazole susceptibility of Haemophilus influenzae (34), we investigated whether it applied to S. aureus.

TSB was inoculated with various concentrations of S. aureus WT or *rexB*::Tn mutant, and growth was assessed in the absence or presence of co-trimoxazole (4 μg ml^−1^). As described above, the growth of S. aureus WT at high bacterial densities was almost completely unaffected by the presence of co-trimoxazole relative to growth medium alone (Fig. 3E and F). However, co-trimoxazole caused a larger degree of growth restriction of S. aureus when lower inoculums were used (Fig. 3E and F). For both the JE2 and SH1000 strains, the inoculum effect was more pronounced for the *rexB*::Tn mutant than for the WT strain (Fig. 3E and F).

The reduced activity of SXT seen under anaerobic conditions suggested that ROS make an important contribution to the bactericidal activity of the antibiotic. Therefore, H_2_DCFDA (2′,7′-dichlorodihydrofluorescein diacetate) dye, which is converted to fluorescent “DCF” in the presence of ROS, was added to growth inhibition assays to quantify and study the kinetics of ROS production during incubation under aerobic conditions (Fig. 3G and I). SXT induced clear concentration-dependent growth inhibition for both the JE2 WT strain and the *rexB* mutant (Fig. 3H and J). When these data were used for normalization to the relative cell density, the results showed that SXT also induced a dose-dependent fluorescent peak at 4 h postinoculation for both WT and *rexBA* mutant strains in the JE2 background (Fig. 3G and I), indicative of the generation of ROS in response to the antibiotic. A second peak in ROS production after 13 h is apparent at lower concentrations of SXT as cells reach the stationary phase, which is likely a result of the bacterial starvation response, which is associated with increased endogenous oxidative stress (35).

Combined, these data provide evidence that SXT induces ROS, which contribute to both the growth-inhibitory activity and the bactericidal activity of the drug. Furthermore, these findings provide evidence that SXT triggers similar levels of oxidative stress in both the WT and the *rexB* mutant, indicating that the increased susceptibility of the mutant to the antibiotic is due to an inability to repair damage rather than to increased generation of ROS.

### RexAB is required for SXT-triggered induction of the SOS response.

The SOS response represents a DNA damage-inducible regulon that includes several genes that encode DNA repair components and is regulated by RecA and LexA (17). Consistent with our findings indicating that SXT causes DNA damage in S. aureus, previous work has shown that TMP triggers *recA* expression, which is indicative of induction of the SOS response (14). This is important because the SOS response increases the mutation rate, promoting the likelihood of the emergence of drug resistance, which is a frequent limitation of SXT therapy (17, 36–38).

**FIG 4:**
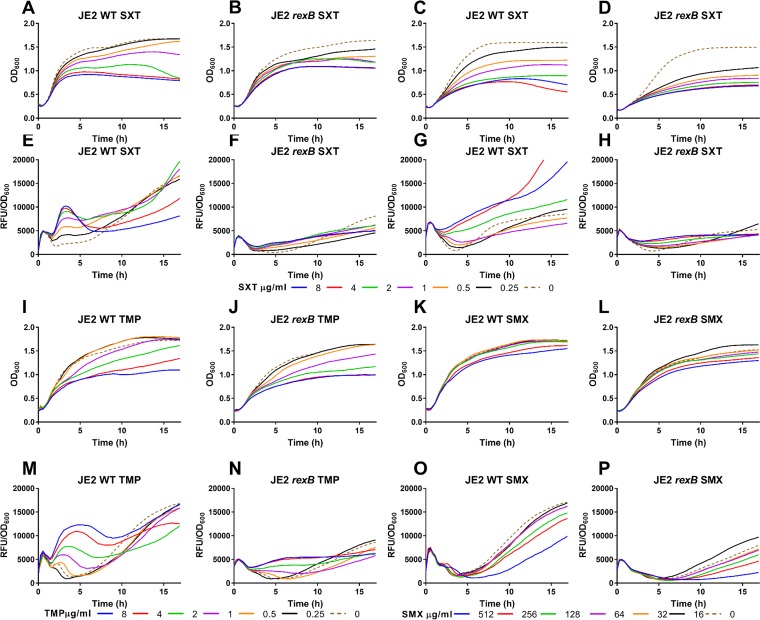
RexAB is essential for the initiation of the SOS response in response to SXT. (A to D and I to L) Growth (OD_600_) of WT JE2 (A, C, I, and K) and the *rexB* mutant (B, D, J, and L) in low-thymidine TSB (A, B, and I to L) and high-thymidine MHB (C and D) in the presence of a range of SXT (A to D), TMP (I to J), and SMX (K and L) concentrations. (E to H) Expression of *recA-gfp* in response to SXT concentrations in S. aureus JE2 WT (E and G) or the *rexB* mutant (F and H) in TSB (E and F) or MHB (G and H). (M to P) Expression of *recA-gfp* in response to TMP (M and N) and SMX (O and P) concentrations in JE2 WT (M and O) or the *rexB* mutant (N and P) in TSB. Fluorescence generated by GFP was measured and normalized to OD_600_ readings to relate *recA* expression levels to cell numbers., In all panels, graphs represent mean values (*n* = 3). Error bars were omitted for clarity.

Since RexAB was important for the repair of DNA damage caused by SXT, we wanted to determine how the complex affected the induction of the SOS response. To investigate this, we used S. aureus JE2 WT and *rexB* mutant strains containing a *recA-gfp* reporter construct (p*recA-gfp*) to measure induction of the SOS response (Fig. 4).

Irrespective of the thymidine concentration, SXT induced dose-dependent inhibition of growth for both the JE2 WT strain and the *rexB* mutant (Fig. 4A to D). In low-thymidine TSB, SXT exposure resulted in a dose-dependent increase in green fluorescent protein (GFP) signal from the p*recA-gfp* reporter construct in the S. aureus WT strain (Fig. 4E), which peaked at about 4 h and then subsided before a the signal slowly increased once more as the bacteria entered stationary phase, presumably due to SOS induction in response to internal DNA-damaging events as a consequence of nutrient limitation and other metabolic stresses (35, 39).

The temporal profile of *recA* expression is similar to that observed for ROS production by WT S. aureus in response to SXT (Fig. 3G), providing additional evidence that antibiotic-induced oxidative stress contributes to DNA damage. However, although SXT also triggered ROS production in the *rexB* mutant (Fig. 3H), the antibiotic did not induce *recA* expression in this strain (Fig. 4F), revealing that RexAB is required for activation of the SOS response under these conditions.

In contrast to experiments done in TSB, *recA* was expressed only weakly during exposure to SXT in high-thymidine MHB (Fig. 4D), which is in keeping with the reduced activity of the antibiotic in this medium (Fig. 2E and F). Consistent with the data from studies in TSB, there was a lack of *recA* expression in the JE2 *rexB* mutant in response to SXT in MHB (Fig. 4E).

Next, we investigated whether SOS induction was due to a single component of co-trimoxazole or to the combination of the two antibiotics. Therefore, S. aureus JE2 strains were exposed to a range of concentrations of trimethoprim or sulfamethoxazole sufficient to cause dose-dependent reductions in growth (Fig. 4I to L). Analysis of *recA* expression revealed that trimethoprim caused a dose-dependent induction of the SOS response in WT S. aureus JE2 but not in the *rexB*::Tn mutant (Fig. 4M and N). In contrast, sulfamethoxazole did not trigger the SOS response at any of the concentrations examined in either the WT or the *rexB*::Tn mutant (Fig. 4O and P). Therefore, it appears that trimethoprim is both necessary and sufficient to trigger the SOS response induced by co-trimoxazole.

### RexAB is required for SXT-induced mutagenic DNA repair.

Derepression of the SOS regulon leads to expression of the error-prone translesion DNA polymerase UmuC, which catalyzes the process of mutagenic DNA repair that contributes to the acquisition of antibiotic resistance (17, 20).

**FIG 5:**
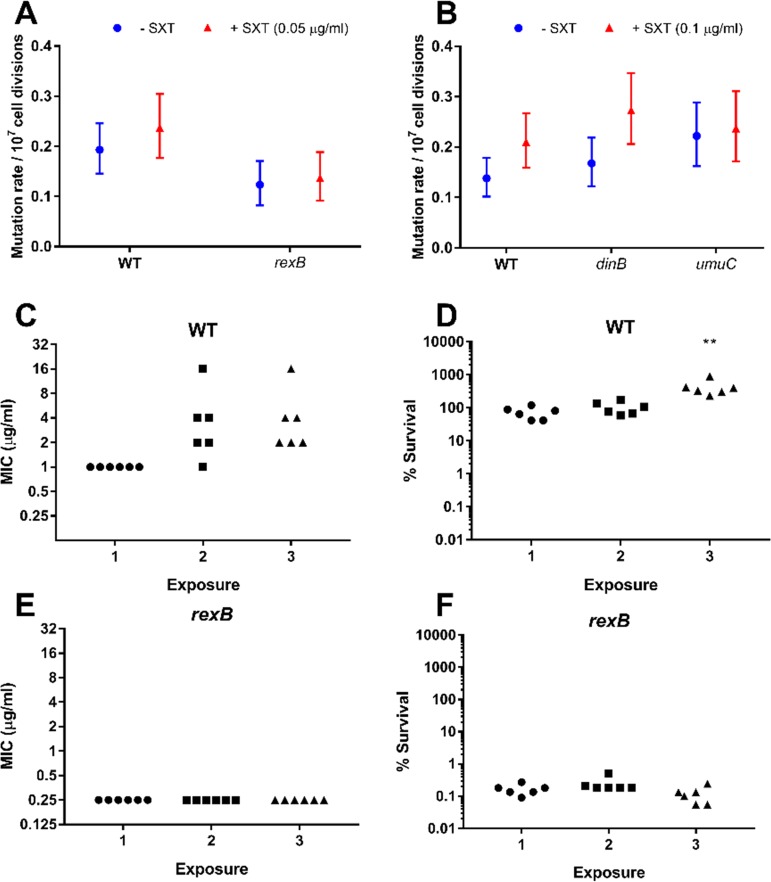
RexAB and UmuC are required for the increased mutation rate mediated by SXT. (A and B) The mutation rate of S. aureus JE2 or mutants lacking components of DNA repair in TSB in the absence or presence of 0.05 μg ml^−1^ (A) or 0.1 μg ml^−1^ (B) SXT. Error bars represent 95% confidence intervals. (C to F) MIC values (C and E) and survival rates (D and F) and of 6 independent cultures of JE2 WT (C and D) and *rexB* mutant (E and F) strains upon 3 rounds of SXT exposure at 4 μg ml^−1^ for 8 h followed by ∼16 h of recovery in TSB. Percent survival at 8 h was calculated relative to the starting inoculum of ∼1 × 10^8^ CFU ml^−1^. Statistical significance was determined by one-way ANOVA corrected for multiple comparisons using the Dunnett method. *, *P ≤* 0.05; **, *P ≤* 0.01; ***, *P ≤* 0.001; ****, *P ≤* 0.0001.

To assess whether SXT promoted SOS-dependent mutagenesis in S. aureus, JE2 WT and DNA damage repair mutants were exposed to subinhibitory concentrations of SXT in low-thymidine TSB. The resulting mutation rate was calculated by fluctuation analysis on the basis of the emergence of rifampin resistance, which occurs via point mutations in the *rpoB* gene encoding the RNA polymerase β-subunit (40, 41). Since the *rexBA* mutant was more susceptible to SXT treatment than the wild-type strain, only a very low concentration of the antibiotic (0.05 μg ml^−1^, 0.2× MIC of the mutant) could be used in mutation rate analyses with this strain. However, even at this concentration, SXT treatment resulted in an increase in the mutation rate of WT S. aureus (Fig. 5A). In contrast to the WT, SXT exposure did not increase the mutation rate of the *rexB* mutant, consistent with the inability of the antibiotic to trigger induction of the SOS response in this strain (Fig. 4B; see also Fig. 5A).

To determine whether the SXT-mediated increase in the mutation rate was due to induction of the SOS response, we exposed the WT and mutants lacking *umuC* or *dinB* (encoding an error-prone polymerase that is not part of the SOS response in S. aureus [17]) to SXT at 0.2× MIC of the mutants (0.1 μg ml^−1^). Consistent with the *recA* reporter data, the higher concentration of SXT used in these experiments promoted the mutation rate of the WT strain to a greater extent than the lower dose (Fig. 4A; see also Fig. 5A and B). SXT caused a similar increase in the mutation rate of the *dinB* mutant but had no effect on the mutation rate of the *umuC* mutant, providing further evidence that co-trimoxazole-mediated SOS induction leads to mutagenic DNA repair (Fig. 5B).

To explore whether RexAB might contribute to the emergence of co-trimoxazole resistance, we exposed 6 independent cultures of the JE2 WT or the *rexB*::Tn mutant to 3 rounds of exposure to the combination antibiotic, followed by a recovery period in TSB alone. After two rounds of exposure to co-trimoxazole, the MIC of 5 of the WT cultures had increased (to 2-fold to 16-fold above that measured for the untreated WT) (Fig. 5C). This was associated with an increased level of WT survival during the third exposure to co-trimoxazole (Fig. 5D). In contrast, there was no change in the co-trimoxazole MIC after 3 rounds of antibiotic exposure, and there was no increase in bacterial survival after 8 h of exposure to the combination antibiotic (Fig. 5E and F).

Taken together, these data demonstrate that SXT exposure results in RexAB-dependent induction of the SOS response in S. aureus, which triggers UmuC-dependent mutagenic DNA repair that increases the rate at which antibiotic resistance arises. In keeping with these findings, RexAB appears to be important for the emergence of SXT resistance *in vitro*.

## DISCUSSION

Multidrug resistance in S. aureus is a major global health concern associated with high treatment failure rates (42). Many second-line or third-line treatments such as vancomycin or daptomycin can lack efficacy require intravenous administration and are associated with toxicity (12). In contrast, SXT is a safe and orally administered antibiotic that is effective in the treatment of skin and soft tissue infections caused by MRSA (4). However, the efficacy of this antibiotic is limited by the ability of the pathogens to bypass SXT-mediated inhibition of folate biosynthesis, either via the uptake of metabolites from the environment or by the frequent acquisition of resistance-conferring mutations (9). Therefore, it is hoped that improved understanding of SXT and its mechanism of action will enable the development of novel approaches aimed at expanding the therapeutic use of this antibiotic and reducing the frequency at which resistance emerges.

The bactericidal effect of SXT has been ascribed to DNA damage, which leads to the induction of the SOS response in bacterial cells (14, 15). The data we present here support the idea that DNA damage is a central component of the mechanism of SXT bactericidal activity in S. aureus and highlight the key role of RexAB in combatting this genotoxicity. Mutants defective for *rexB* were significantly more susceptible to SXT than the WT in both growth inhibition and killing assays.

It should be noted, however, that the increased susceptibility of the *rexB* mutants to co-trimoxazole, relative to the wild type, was much more apparent in killing assays than in those that measured growth inhibition (MIC and zone of inhibition). This may indicate that the genotoxicity of co-trimoxazole is dose dependent, a conjecture which is supported by data showing that both ROS generation and *recA* expression increase with the dose of the combination antibiotic. Fortunately, the low toxicity of co-trimoxazole enables high doses to be used therapeutically, with mean serum concentrations above the breakpoint (43).

RexAB is proposed to be a member of the AddAB family of DNA repair enzymes required for the processing of DNA double-strand breaks on the basis of protein sequence identity (19, 20, 44). Therefore, the increased susceptibility of *rexB* mutants to SXT indicates that DNA DSBs are caused by the antibiotic and are lethal if not repaired. Additional evidence that SXT causes DNA damage in S. aureus came from analysis of the *recF* mutant, which was more susceptible to the antibiotic than the WT, albeit to a lesser extent than the *rexB* mutant. The function of RecF has been ascribed to the repair of DNA lesions that arise from DNA single-strand gaps. However, while this function is well established in canonical model organisms, including E. coli and Bacillus subtilis, for bacterial DNA damage repair, the evidence that supports RecF performing a similar function in S. aureus is less strong (18). Therefore, a greater understanding of how RecF repairs damage in staphylococci would provide insight into the type of DNA damage caused by SXT in S. aureus.

Apart from *rexA* and *rexB* mutants, none of the other strains lacking DNA repair components had increased SXT susceptibility. While it is possible that SXT causes DNA damage beyond DSBs, those other types of damage do not appear to contribute to growth inhibition or bacterial killing. This finding also implies that DNA repair does not contribute to the lethality of SXT, as has also been reported for trimethoprim-mediated killing of E. coli (13).

The discovery that S. aureus is more susceptible to SXT under conditions of incubation in thymidine-poor medium is in keeping with previous reports which suggested that thymidine depletion makes a crucial contribution to the lethality of SXT (15, 30). Specifically, nucleotide imbalance, caused by thymidine depletion, can result in the collapse of replication forks during DNA synthesis, which can lead to DNA DSBs (8). In support of this, we observed SOS induction under thymidine-limited but not thymidine-replete conditions. This is thought to have important implications for the efficacy of SXT treatment. While the concentration of free thymidine in plasma is relatively low (∼0.2 μM/∼48 ng ml^−1^) (45), elevated thymidine concentrations are likely to occur in human tissues containing necrotic cells or neutrophil extracellular traps (9, 46). To acquire this thymidine, S. aureus expresses DNase to break down DNA and NupC, a nucleoside transporter that has been shown to support the growth of S. aureus during SXT exposure by enabling the uptake of extracellular thymidine (47, 48).

The presence in host tissues of thymidine and other folate-dependent metabolites, including serine, methionine, and glycine, has been reported previously to modulate the susceptibility of SXT-treated cells and restrict the types of infection that can be treated with this antibiotic (9). However, while the presence of thymidine reduced the susceptibility of the WT strain to SXT, there was still a >1,000-fold reduction in CFU counts of the *rexB* mutant exposed to the antibiotic, suggesting that thymidine limitation is not solely responsible for DNA damage in S. aureus (Fig. 2G and I).

In agreement with several reports that bactericidal antibiotics, including TMP, exert part of their antibacterial effect via the generation of ROS (13, 49), we determined that the bactericidal effect of SXT against S. aureus is dependent upon the availability of oxygen. This was evidenced by the complete lack of bacterial killing under anaerobic conditions and suggests that ROS-mediated DNA damage makes a greater contribution to the bactericidal activity of SXT than the inhibition of DNA synthesis caused by thymidine depletion under aerobic conditions. However, it should also be considered that bacterial replication and physiology under anaerobic conditions differ significantly from the replication and physiology seen under aerobic growth conditions. Therefore, future work will attempt to further define the role of ROS in SXT-mediated killing, for example, by testing whether SXT exposure under aerobic conditions leads to oxidative DNA damage. Antibiotic-induced oxidative stress has been shown to increase the intracellular level of 8-oxodeoxyguanosine (8 -oxo-dG), which is incorporated into DNA and paired with deoxycytidine or deoxyadenosine (50). Incomplete repair of DNA-incorporated 8-oxo-dG can lead to DSBs in E. coli, which contributes to the lethality of TMP (13). Additional experiments using mutants defective for the repair of 8-oxo-dG will determine whether a similar mechanism mediates the genotoxicity of SXT in S. aureus under aerobic conditions. However, while ROS appear to make an important contribution to the bactericidal activity of SXT, the increased susceptibility of the *rexB* mutant to this antibiotic under anaerobic conditions confirms that DNA damage can occur independently of ROS. This may explain why there are phenotypic differences in the effects of SXT exposure and thymineless death observed in *thyA* mutants deprived of thymine (13).

One drawback of SXT is the frequent emergence of resistance during long-term therapy (51). In keeping with the induction of the SOS response, exposure of S. aureus to subinhibitory concentrations of SXT was found to increase the mutation rate via the activity of UmuC, a low-fidelity DNA polymerase, previously shown to mediate mutagenic DNA repair of bacteria exposed to ciprofloxacin or H_2_O_2_ (20). Similarly to what has been reported for ciprofloxacin and H_2_O_2_, SXT-induced increases in the mutation rate were dependent upon the presence of RexAB (20). Therefore, the processing of DNA DSBs by RexAB is central to the survival of S. aureus exposed to SXT as well as to SOS-mediated increases in the mutation rate associated with drug resistance.

In summary, our data demonstrate that SXT causes DNA damage in S. aureus via thymidine depletion and ROS generation, the repair of which via RexAB enables bacterial survival and induction of the SOS response and appears to contribute to the emergence of drug resistance *in vitro*. The identification of RexAB as central to both SXT susceptibility and SOS induction suggests that this protein complex may provide a target for novel therapeutic approaches that could improve the efficacy of SXT and reduce the emergence of resistance. Such an approach may also increase the range of MRSA infections that could be treated with SXT.

## MATERIALS AND METHODS

### Bacterial strains and culture conditions.

A full list of the bacterial strains used in this study is provided in Table 1. S. aureus was cultured in either tryptic soy broth (TSB) or Mueller-Hinton broth (MHB) supplemented with calcium (25 mg/liter) and magnesium (12.5 mg/liter) to the stationary phase for 18 h at 37°C, with shaking (180 rpm). Culture media were supplemented with erythromycin (10 μg ml^−1^) for growth of transposon mutants, kanamycin (90 μg ml^−1^) for growth of the *recA* reporter strains, or anhydrotetracycline hydrochloride (AHT) (10 or 100 ng ml^−1^) to induce expression of *rexBA* on plasmids, as required (Table 1).

**TABLE 1:** Bacterial strains used in this study

Bacterial strain	Relevant characteristic(s)^*a*^	Reference or source
Methicillin-resistant S. aureus		
USA300 LAC JE2	LAC strain of the USA300 CA-MRSA lineage cured of plasmids	60
USA300 LAC JE2 *rexB*::Tn	USA300 LAC JE2 with a *bursa aurealis* transposon insertion in *rexB*, Ery^r^	60
USA300 LAC JE2 *umuC*::Tn	USA300 LAC JE2 with a *bursa aurealis* transposon insertion in *umuC*, Ery^r^	60
USA300 LAC JE2 *recF*::Tn	USA300 LAC JE2 with a *bursa aurealis* transposon insertion in *recF*, Ery^r^	60
USA300 LAC JE2 *dinB*::Tn	USA300 LAC JE2 with a *bursa aurealis* transposon insertion in *dinB*, Ery^r^	60
USA300 LAC JE2 *rexB*::Tn p*itet* empty	USA300 LAC JE2 with a *bursa aurealis* transposon insertion in *rexB* with integrated p*itet* empty plasmid, Ery^r^	This study
USA300 LAC JE2 *rexB*::Tn p*itet rexB*	USA300 LAC JE2 with a *bursa aurealis* transposon insertion in *rexB* with integrated p*itet* with AHT-inducible *rexB*, Ery^r^	This study
USA300 LAC JE2 +pCN34 *recA-gfp*	USA300 LAC JE2 containing pCN34 with *gfp* under the control of the *recA* promotor, Kan^r^	This study
USA300 LAC JE2 *rexB*::Tn +pCN34 *recA-gfp*	USA300 LAC JE2 with a *bursa aurealis* transposon insertion in *rexB* containing pCN34 with *gfp* under the control of the *recA* promotor, Kan^r^	This study
USA300 LAC JE2 +pCN34 Empty	USA300 LAC JE2 containing pCN34 empty vector, Kan^r^	This study
USA300 LAC JE2 *rexB*::Tn +pCN34 Empty	USA300 LAC JE2 with a *bursa aurealis* transposon insertion in *rexB* containing pCN34 empty vector, Kan^r^	This study

Methicillin-sensitive S. aureus		
SH1000	Methicillin sensitive S. aureus strain	61
SH1000 *rexB*::Tn	SH1000 with a *bursa aurealis* transposon insertion in *rexB*, Ery^r^	20
SH1000 *rexB*::Tn p*itet* Empty	SH1000 with a *bursa aurealis* transposon insertion in *rexB* with integrated p*itet* empty plasmid, Ery^r^	This study
SH1000 *rexB*::Tn p*itet rexB*	SH1000 with a *bursa aurealis* transposon insertion in *rexB* with integrated p*itet* with AHT-inducible *rexB*, Ery^r^	This study
	SH1000 containing pCN34 with a *gfp* under the control of the *recA* promotor, Kan^r^	This study

a Ery^r^, erythromycin resistance; Kan^r^, kanamycin resistance.

S. aureus SH1000 and JE2 mutants deficient for *rexB* were complemented using p*itet* (52) containing the wild-type *rexBA* operon. Since insertion of the transposon into *rexB* is expected to block the expression of the downstream *rexA* gene, the *rexB* mutants were complemented with the entire *rexBA* operon. The p*itet* vector is a single-copy plasmid that integrates into the *geh* locus of the S. aureus genome. It contains a tetracycline-inducible promoter that was used to control *rexBA* gene expression. Primers containing the AvrII or PmeI restriction sites (Table 2) were used to amplify the *rexBA* operon from USA300 JE2 genomic DNA. The PCR product and p*itet* vector were digested using AvrII and PmeI restriction enzymes, ligated using T4 ligase, and transformed into CaCl_2_-competent E. coli DH5α cells. Successful ligation of *rexBA* into p*itet* was confirmed with DNA sequencing. Plasmid DNA was subsequently transformed into the DC10B E. coli strain, which lacks cytosine methylation, to allow bypassing of the staphylococcal restriction-modification barrier (53), followed by successful electroporation into electrocompetent S. aureus *rexB* mutants in the JE2 and SH1000 genetic backgrounds. Empty vector that did not contain the *rexBA* operon was used as a control. Successful integration into the S. aureus genome was confirmed by PCR amplification of the *geh* gene followed by DNA sequencing.

**TABLE 2:** Primers used for construction of complemented *rexB* mutants and *recA-gfp* reporter

Oligonucleotide	Sequence (5′–3′) (restriction site underlined)
Primers used for construction of *rexB*::Tn p*rexBA* complemented mutant	
*rexB*-F AvrII	GGCCCTAGGATGACATTACATGCTTATTTAG
*rexA*-R PmeI	GCCGTTTAAACCTATAGTTGCAATGTACCAAATTTG
pCL55 Fwd seq	GGATCCCCTCGAGTTCATG
pCL55 Rev seq	CTCGTAGTATCTATACTTCG
Lipase *geh* F	GTTGTTTTTGTACATGGATTTTTAG
Lipase *geh* R	CTTGCTTTCAATTGTGTTCC
pCL55 R	GCGCATAGGTGAGTTATTAGC

Primers used for construction of the *recA-gfp* reporter	
P*recA*-F BamHI	GAGGATCCTATGGTTCAGATGACACAT
P*recA*-R 7xT-GFP	CATTTTTTTTCCTCCTAATTGAAATTGC
GFP-F 7xA-P*recA*	AGGAAAAAAAATGAGTAAAGGAGAAGAACT
GFP-R KpnI	GCGGGTACCTTATTTGTATAGTTCATCCATG
pCN34 seq F	GTTATCCCCTGATTCTGTGGATAAC
pCN34 seq R	CCAGAATTATATTCAGAACAGGAAC

### MIC assay.

The MIC was determined according to a previously described broth microdilution protocol (54). Briefly, 95-μl aliquots of either MHB or TSB were supplemented with doubling concentrations of co-trimoxazole (SXT), trimethoprim (TMP), or sulfamethoxazole (SMX) ranging from 0.008 μg ml^−1^ to 512 μg ml^−1^ in 96-well plates. In some assays, the culture medium was also supplemented with thymidine. In this study, the concentration of SXT was calculated based on the concentration of TMP present within the combination therapeutic. Stationary-phase cultures of S. aureus were diluted and inoculated into the antibiotic-containing media to reach a final cell density of 1 × 10^5^ CFU ml^−1^. For complemented mutants containing *rexBA* under the control of the p*itet* promoter, the medium was supplemented with AHT (100 ng ml^−1^) to induce gene expression. Plates were incubated for 17 h at 37°C before readings were performed an optical density at 600 nm (OD_600_). The MIC was determined as the lowest concentration of antibiotic required to cause growth inhibition. To determine MICs under anaerobic conditions, 96-well plates were incubated in anaerobic jars containing Anaerogen gas packs at 37°C for 18 h.

### SXT killing assay.

Stationary-phase S. aureus cultures were washed twice by alternating rounds of centrifugation and resuspension to a density of 2 × 10^8^ CFU ml^−1^ in fresh MHB or TSB (3 ml) containing 4 μg ml^−1^ SXT, representing the antibiotic clinical breakpoint concentration (55). Some experiments investigating the inoculum effect used lower starting bacterial cell densities, such as ∼10^7^, 10^6^, and 10^5^ CFU ml^−1^. Bacteria were incubated with SXT at 37°C with shaking (180 rpm), and rates of survival were determined over time by quantification of CFU using the method of Miles et al. (56), which consists of serial dilution followed by plating onto agar plates to enable the enumeration of colonies. Data were converted to percent survival by comparing CFU counts at specific time points with the starting inoculum. To investigate SXT-mediated killing under anaerobic conditions, washed stationary-phase cultures were inoculated into prereduced broth in bijou tubes supplemented with 4 μg ml^−1^ SXT and were then incubated statically at 37°C with the vessel caps loosened in an anaerobic jar containing an AnaeroGen gas pack. The growth of S. aureus strains in TSB under aerobic or anaerobic conditions was examined using a similar protocol and TSB or MHB without antibiotics.

### Disc diffusion assay.

Stationary-phase S. aureus cultures were adjusted to an OD_600_ of 0.063 (0.5 McFarland) in TSB and used to inoculate tryptic soy agar (TSA) plates using a cotton wool swab. Antibiotic discs containing 2.5 μg of either SXT or TMP (Oxoid) were placed onto the inoculated agar and incubated at 37°C under aerobic or anaerobic conditions and incubated at 37°C for 17 h. The zone of inhibition was measured by calculating the total diameter of the cleared bacterial lawn minus the diameter of the antibiotic disc.

### Mutation rate analyses.

Mutation rates were determined as described previously (20, 40). Briefly, 30 parallel cultures of S. aureus were used for each condition or strain in bijou tubes containing 1 ml TSB inoculated with S. aureus at ∼5 × 10^5^ CFU ml^−1^ and incubated with or without 0.05 μg ml^−1^ (0.1× wild-type MIC) or 0.1 μg ml^−1^ (0.2× wild-type MIC) SXT with shaking at 37°C for 24 h. After incubation, 10 cultures were selected at random, and total CFU counts were determined through serial dilution in phosphate-buffered saline (PBS) followed by plating onto TSA plates. Subsequently, 100 μl of each of the 30 undiluted cultures was spread on TSA plates containing rifampin (100 μg ml^−1^) before incubation at 37°C for 24 h. The number of rifampin-resistant colonies on each plate was counted, and the mutation rate was calculated with 95% confidence intervals using the maximum-likelihood setting of the FALCOR mutation rate calculator (40). Since these assays were done in the presence of SXT, it was important to use rifampin resistance (rather than SXT resistance) to measure the mutation rate to avoid selecting for resistance emergence. Rifampin resistance is a well-established marker for mutation rate analysis and is not selected for or against under the conditions used (20, 40).

### *In vitro* SXT resistance emergence assay.

This assay was based on that described previously for the emergence of daptomycin resistance (57). S. aureus was inoculated to ∼10^8^ CFU ml^−1^ in 3 ml TSB containing co-trimoxazole (4 μg ml^−1^) for 8 h per exposure. After that time, bacterial survival was determined by CFU counts, which were compared to the inoculum. For repeated antibiotic exposures, 1 ml of each culture was centrifuged (3 min, 17,000 × *g*) and the resulting pellet washed once in PBS to remove the antibiotic. The pellet was resuspended in 100 μl TSB, and ∼10^5^ CFU (based on survival data) was used to inoculate 3 ml TSB before incubation for 16 h at 37°C with shaking (180 rpm) in the absence of antibiotics to enable bacterial recovery. Co-trimoxazole MIC assays were undertaken on these cultures to detect changes in susceptibility. Recovered bacterial cultures were then reexposed to co-trimoxazole for a total of three repeated exposures.

### *recA* reporter assay.

Induction of the SOS response in S. aureus in response to exposure to TMP and SMX and to the SXT combination was determined through the use of strains containing a *recA-gfp* reporter construct. These were generated by transforming S. aureus with pCN34 containing the *recA* promoter upstream of *gfp*. Primers (detailed in Table 2) were used to amplify the *recA* promoter region (sequence detailed in reference 14) from JE2 wild-type genomic DNA and the *gfp* gene in the pCL55 P3 GFP plasmid (58). These products were combined by overlapping extension PCR using primers PrecA-F BamHI and GFP-R KpnI, digested with BamHI and KpnI (relevant restriction sites incorporated into amplicon primers), and ligated into phosphatase-treated, BamHI- and KpnI-digested low-copy-number shuttle vector pCN34. Empty vector without the *recA-gfp* construct served as a control. The *recA-gfp* pCN34 vectors were constructed in E. coli DC10B and confirmed by sequencing. Cultures of DC10B containing the *recA-gfp* pCN34 vector and empty vector were cultured in LB broth supplemented with ampicillin (100 μg/ml) to select for plasmid maintenance. Using methods described in reference 53, *recA-gfp* and empty vectors were transformed directly into S. aureus WT JE2 and the *rexB*::Tn mutant. Reporter strains were grown in the presence of 90 μg/ml of kanamycin to maintain the plasmid.

Stationary-phase cultures of *recA-gfp* reporter strains were pelleted by centrifugation, washed twice, and resuspended in TSB supplemented with kanamycin (90 μg ml^−1^). Cultures (∼3.33 × 10^8^ CFU ml^−1^) were exposed to a range of TMP, SMX, and SXT concentrations (0.01 μg m^−1^ to 512 μg ml^−1^) in black-walled microtiter plates. These were incubated, with shaking, at 37°C for 17 h in a TECAN Infinite 200 PRO microplate reader, where OD_600_ and GFP relative fluorescence units (RFU) (excitation, 475 nm; emission, 525 nm) were quantified every 1,000 s (∼17 min). The values were blanked against values generated from uninoculated wells, and GFP fluorescence was normalized to OD_600_ readings to determine *recA* expression levels.

### Endogenous ROS production.

Production of ROS was detected using the permanent cell dye 2’,7’-dichlorodihydrofluorescein diacetate (H_2_DCFDA), which is converted to fluorescent DCF by oxidative cleavage of acetate groups (59). A 96-well microtiter plate assay was prepared using a format similar to that used for the *recA-gfp* reporter assay, with the addition of 25 μM H_2_DCFDA and various concentrations of SXT. Wild-type and *rexB*::Tn mutant strains were washed and inoculated at ∼3.33 × 10^8^ CFU ml^−1^. Plates were incubated with shaking at 37°C as described previously, and OD_600_ and fluorescence (excitation, 495 nm; emission, 525 nm) were quantified every 1,000 s (∼17 min). OD_600_ values were normalized against uninoculated wells, and fluorescence data were normalized against untreated wells. Fluorescent values were normalized to OD_600_ readings to determine levels of production of ROS relative to cell number. Bacterial growth rates (μ, expressed as ΔOD_600_ min^−1^) were calculated for logarithmic-phase growth using the following formula: μ = (ln OD_2_ – Ln OD_1_)/(*t*_2_ – *t*_1_).

### Statistical analysis.

Means or medians were calculated from at least three biologically independent replicates, and the data were analyzed by Student's *t* test (two-tailed, unpaired, assuming equal variances), One-way analysis of variance (ANOVA) or two-way ANOVA corrected for multiple comparisons was performed using GraphPad Prism (V7.0) as described in the figure legends. MIC bar graphs show median values. All remaining graphs were plotted to show means ± standard deviations (SD). Error bars were omitted on *recA* reporter data for clarity.

Data were not log transformed, with the exception of growth rate analyses.
